# Oxygenation and Hemodynamics Do Not Underlie Early Muscle Fatigue for Patients with Work-Related Muscle Pain

**DOI:** 10.1371/journal.pone.0095582

**Published:** 2014-04-22

**Authors:** Guilherme H. Elcadi, Mikael Forsman, David M. Hallman, Ulrika Aasa, Martin Fahlstrom, Albert G. Crenshaw

**Affiliations:** 1 Centre for Musculoskeletal Research, University of Gävle, Gävle, Sweden; 2 Institute of Environmental Medicine, Karolinska Institutet, Stockholm, Sweden; 3 Department of Community Medicine and Rehabilitation, Rehabilitation medicine, Umeå University, Umeå, Sweden; 4 Department of Clinical Sciences, Professional Development, Umeå University, Umeå, Sweden; 5 Department of Community Medicine and Rehabilitation, Physiotherapy, Umeå University, Umeå, Sweden; University of South Australia, Australia

## Abstract

Patients suffering from work-related muscle pain (WRMP) fatigue earlier during exercise than healthy controls. Inadequate oxygen consumption and/or inadequate blood supply can influence the ability of the muscles to withstand fatigue. However, it remains unknown if oxygenation and hemodynamics are associated with early fatigue in muscles of WRMP patients. In the present study we applied near-infrared spectroscopy (NIRS) on the extensor carpi radialis (ECR) and trapezius (TD) muscles of patients with WRMP (n = 18) and healthy controls (n = 17). Our objective was to determine if there were group differences in endurance times for a low-level contraction of 15% maximal voluntary contraction (MVC) – sustained for 12–13 min, and to see if these differences were associated with differences in muscle oxygenation and hemodynamics. At baseline, oxygen saturation (StO_2_%) was similar between groups for the ECR, but StO_2_% was significantly lower for TD for the WRMP patients (76%) compared to controls (85%) (*P*<0.01). Also, baseline ECR blood flow was similar in the two groups. For both muscles there were a larger number of patients, compared to controls, that did not maintain the 15% MVC for the allotted time. Consequently, the endurance times were significantly shorter for the WRMP patients than controls (medians, ECR: 347 s vs. 582 s; TD: 430 s vs. 723 s respectively). Responses in StO_2_% during the contractions were not significantly different between groups for either muscle, i.e. no apparent difference in oxygen consumption. Overall, we interpret our findings to indicate that the early fatigue for our WRMP patients was not associated with muscle oxygenation and hemodynamics.

## Introduction

In addition to localized discomfort, sufferers of work related muscle pain (WRMP) frequently proclaim a sensation of muscle fatigue [1], [2]. In fact, studies have shown that patients with WRMP fatigue earlier during exercise than healthy controls [3]–[6]. An important factor related to fatigue mechanisms of muscles during exercise is the ability to supply and utilize oxygen to and by the muscle tissue. In other words, inadequate blood supply and/or inadequate oxygen consumption can influence the ability of the muscles to withstand fatigue [7], [8]. However, studies using objective measurements of hemodynamics or oxygenation to investigate their involvement in the fatigue for patients with WRMP are sparse.

In a number of studies, fatigue onset and recovery have been assessed for laboratory models of low-level contractions [9]–[11] because this work model simulates the type of work (e.g. computer work, assembly line work) associated with developing WRMP. An implied intent in these studies is to shed light on the underlying mechanism behind WRMP. Practically all previous studies have employed healthy subjects, and findings of an immediate onset of fatigue followed by a prolonged effect lasting up to hours after the end of exercise have been reported [9]. We propound that by studying WRMP patients in these contexts, and by including relevant objective methods, our understanding of the mechanism of early muscle fatigue and persistent pain for this group could be enhanced.

Near-infrared spectroscopy (NIRS) is a non-invasive technique that allows for the continuous monitoring of changes in local muscle oxygenation. With experimental manipulation several other important physiological variables can be appreciated (c.f. Crenshaw *et al*. [12]). These include re-oxygenation rate, oxygen recovery, blood flow, oxygen consumption and oxygen delivery. Thus, NIRS appears useful to provide relevant physiological data for assessing muscle fatigue for WRMP patients. As far as we know, there is only one study of this type. Kell and Bhambhani [13] showed disturbed muscle oxygenation and slower oxygenation recovery following a fatigue test for a group of subjects with low back pain compared to healthy controls. However, the same study showed no differences in time to fatigue between patients and controls, and attributed factors other than aerobic capacity to underlie endurance capacity.

There are NIRS studies (not focusing on fatigue) that compared responses between healthy controls and patients with WRMP for low-level work. However, the results of these studies have been somewhat conflicting. Brunnekreef and co-workers [14] reported a decrease in oxygen consumption and reduced blood flow in patients with WRMP in the forearm after isometric contractions. Sjogaard *et al*. [15] claimed that oxygenation saturation patterns in the shoulder during pegboard work differed between patients with WRMP and healthy subjects as there was a trending increase toward recovery during the work for the healthy subjects that did not occur for the patients. However, Flodgren *et al*. [16] did not see a difference in shoulder oxygen saturation patterns between patients and healthy controls performing repetitive arm work. Furthermore, Elcadi *et al*. [17] showed no differences between patients and controls for oxygen use, recovery, and blood flow during and after short-term isometric contractions and recovery. Differences in experimental settings, body regions, and choice of NIRS variables may account for discrepancies between studies.

The primary aim of the study was to determine if there are differences in endurance times between WRMP patients and healthy controls for a low-level sustained contraction (15% MVC), and if so to determine if these differences are associated with group differences in oxygenation and hemodynamics. To elucidate on previous findings, a secondary aim was to compare oxygenation responses for the two groups during the sustained contraction, and present these data together with electromyography (EMG) and electrocardiography (EKG) data.

## Materials and Methods

### Participants – Patients and healthy control subjects

Eighteen patients with work related muscle pain, consisting of four males and 14 females, participated in the present study. Patients were referred to us by three occupational health clinics in Umeå, Sweden (see table 1 for personal data). All patients were identified at the occupational health clinics as suffering from work related neck/shoulder and/or forearm muscle pain. Inclusion criteria were: Patients of 20–65 years old, suffering from work-related nociceptive pain or discomfort in the right side of the neck/shoulder and/or forearm for a minimum time of three months. Patients were excluded if they were on medication that impacted on circulation, autonomic responses or heart rate, or if they had peripheral or central neuropathic pain. For diagnosis confirmation at the time of the study, all patients were independently screened by two trained physiotherapists (one a co-author, UA) who are expert in diagnosis of work related muscle pain. The patients had experienced pain in both the ECR and TD muscle regions from 6 months to 13 years (median 6 yrs), and their occupations consisted of office workers (n = 10), dental hygienists (n = 5) and factory workers (n = 3), and all were currently working. Patients with bilateral pain were also included; however, it was crucial for our study equipment that patients had been diagnosed with pain on the right side. Approximately 70% of all patients sent to us were included in the study.

**Table 1:** Anthropometric and personal data for patients and controls showing age, height, weight, adipose tissue thickness (ATT), muscle thickness (Mth), hemoglobin concentration (Hb cont), rest oxygen saturation for the ECR and TD (Rest ECR and Rest TD), rest ECR blood flow (HbTslope), maximal voluntary contractions for the ECR and TD (MVC ECR and MVC TD), and systolic blood pressure and diastolic blood pressure.

	Patients (n = 18)	Controls (n = 17)	*P*
**Age (yrs)**	43.44 (10.6)	39.00 (12.1)	0.26
**Height (m)**	169.00 (8.4)	169.94 (9.9)	0.76
**Weight (kg)**	72.22 (13.5)	65.50 (11.5)	0.13
**ATT ECR (mm)**	5.58 (1.6)	4.78 (1.6)	0.15
**MTh ECR (mm)**	16.69 (6.1)	15.55 (5.0)	0.55
**ATT TD (mm)**	5.97 (1.2)	4.98 (1.4)	0.04*
**MTh TD (mm)**	11.73 (2.8)	10.35 (2.2)	0.12
**Hb cont (gram/L)**	136.33 (14.0)	138.18 (16.3)	0.72
**Rest ECR (StO_2_%)**	64.39 (12.6)	69.94 (12.5)	0.20
**Rest TD (StO_2_%)**	76.31 (9.4)	85.43 (9.1)	<0.01*
**Rest ECR blood flow (HbTslope)**	0.12 (0.12)	0.15 (0.14)	0.58
**MVC ECR (N)**	151.94 (53.8)	156.82 (44.0)	0.77
**MVC TD (N)**	416.80 (132.0)	454.35 (142.9)	0.45
**Blood Pressure Systolic (mmHg)** †	121.86 (13.9)	126.80 (18.1)	0.20
**Blood Pressure Diastolic (mmHg)** †	80.39 (8.2)	76.85 (8.6)	0.08

Values are means (±SD) unless specified otherwise. ***P*** indicates differences between patients and controls.

*Indicates significance.

† Group mean values of both test occasions.

At the occupational health clinics patients were provided with an information sheet which described the purpose of the research. Patients were matched with a randomly selected group of healthy control subjects comparable to the patient group with regard to age and sex. All controls were in good health and free of discomfort related to the upper body. Prior to testing, all patients and controls were independently screened by the same two physiotherapists. The tests confirmed nociceptive pain for the patient group and no symptoms of chronic or acute pain for the control group. Furthermore, patients and controls completed a questionnaire concerning their level of physical activity and general health. All subjects were right handed. In their majority, both groups reported to be regularly participating in some kind of activity at a gym or training facility. All participants were given verbal and written descriptions of the experimental protocols and signed informed consents prior to the participation in the study. Table 1 shows anthropometric as well as other personal data for patients and controls. The study was approved by the local ethical committee of Uppsala, Sweden (Dnr 2008/268) in accordance to the Declaration of Helsinki on the use of human subjects in research.

### Experimental set-up and protocol

Participants attended the experimental laboratory on two occasions and were asked not to perform any extensive physical activity on the day prior to each test occasion, not to drink coffee or any caffeinated beverage, and to not have a heavy meal two hours prior to each test occasion. On both days, subjects underwent an array of preparation procedures prior to the beginning of the test protocol. Blood pressure measurements were first taken to control for differences between patients and controls. Blood pressure mean levels between days did not differ significantly at the time of the experiment (mean values are given in table 1). Next, on each day depending on the muscle investigated, sites for ultrasound and NIRS measurements were identified for the forearm extensor carpi radialis (ECR) and trapezius descendens (TD) muscles (muscles were alternated for each participant between first and second test occasions). For the ECR the measurement site was 2 cm distal to a point half-way between the supracondylar ridge and the inner bend of the elbow. The site on the TD was one-third the distance (in a lateral direction) between C7 and the acromion process of the scapula. Ultrasound imaging (Aloka SSD-2000, Aloka Co., Ltd., Japan) was then performed at each respective site to attain a measurement of the distance from skin surface to the muscle (i.e. adipose tissue thickness, ATT) and the muscle thickness (MTh). The NIRS probe was placed over the ECR and TD in the direction of the muscle fibers. An adhesive shield with a window was attached to the skin to secure the probe. To calculate and locate placement sites in the same manner for all subjects, the shield was positioned by the same investigator on all occasions.

**Figure 1:**
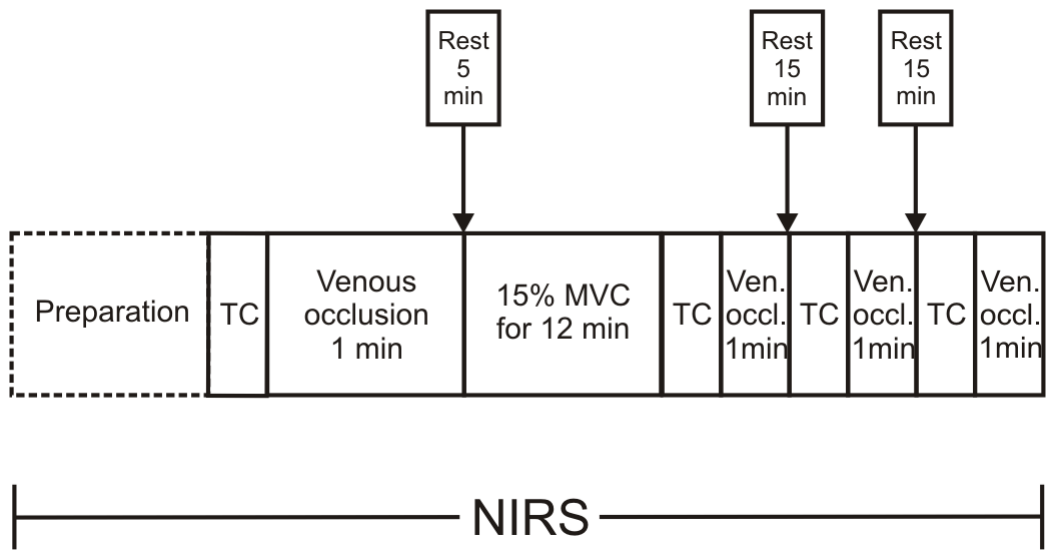
Test Protocol. Experimental protocol for the ECR and TD muscles showing the performances of test contractions (TC), venous occlusions (VO) before and after the sustained contraction, sustained submaximal isometric contraction 15% MVC to a maximum of 12 min. NIRS, EMG and EKG were recorded continuously throughout the experiment.

The overall experimental flow included maximal voluntary contractions (MVC), followed by a test contraction (TC) and a venous occlusion (VO). Subsequently, a submaximal isometric contraction at 15% MVC was performed followed by a TC and a VO immediately after the cessation of the contraction. After resting intervals of 15 min each two more TCs and VOs were applied (Fig. 1).

For MVC and sustained isometric contractions of the ECR, subjects sat comfortably with the back supported in a sturdy upright chair. The forearm was placed on a special designed arm support with the wrist in a pronated position, palm facing downwards, and placed at the edge of a height adjustable table. For each subject the height of the arm support was adjusted to keep the shoulders horizontally lined up avoiding trunk bending sideways. The subjects were instructed to sit in an upright position to obtain an elbow joint angle of ∼100 degrees. A vertical strap was positioned over the dorsum of the hand; the other end of the strap was attached to a strain gauge dynamometer (KKM-1, Bofors, Sweden) that was bolted to the floor next to the chair. The task was to extend the wrist joint in an attempt to pull the strap upwards while producing force. The forearm and wrist remained in contact with the testing table for the whole duration of the procedures, thereby minimizing the action of upper arm and shoulder muscles on the exerted force. For VO, participants were moved to a hospital gurney (Sjöbloms AB, Sweden) and were seated in a reclined position with the forearm at heart level.

For the TD aspect of the experiment, participants were again seated in the testing chair as described above. The arms were positioned vertically along the body so as to minimize the involvement of biceps and deltoid muscles during force production [18]. With the right hand, subjects grasped a handle securing a firm grip (Eleiko – 22 in. handle straps). The handle was connected to a strap attached to the strain gauge dynamometer, also used for the ECR. The same type of strap with handle arrangement was used on the subject's left side but this was attached to a fixture on the floor, i.e. force measurements were only conducted for the right side. Both handles were aligned with straight arms and adjusted at heights that made the shoulders align horizontally. Participants were instructed to produce isometric force by attempting to elevate the shoulder joints on both sides of the body simultaneously.

### Oxygenation measurements

Non-invasive measurements of local tissue oxygenation, deoxyhemoglobin (HHb), oxyhemoglobin (HbO) and total haemoglobin (HbT) using near infrared spectroscopy (NIRS) in the ECR and TD were made using a spectrometer (INSPECTRA Tissue Spectrometer-model 325, Hutchingson Technology Inc., MN, USA) capable of emitting and detecting light intensities at wavelengths 680, 720, 760 and 800 nm [19]. Of note, myoglobin has the same absorbance spectrum as hemoglobin but it absorbs at less than 10%, which is considered negligible [20]. Prior to placing the probe on the body it was inserted in a light scattering calibrator for capturing reference light intensities of all applied wavelengths. All tissue measurements were related to these reference data, thereby converting light intensity measurements to optical absorbance. Optical absorbance values were further processed into a scaled second derivative absorbance spectrum, whereby a measure of the oxygen saturation of haemoglobin was obtained and expressed as percent saturation (StO_2_%). The software supplied with the Inspectra device provides estimated absolute values of StO_2_%. HHb and HbO are also reported and relatively expressed from a preset baseline value as arbitrary units. From these data, total haemoglobin, which reflects the blood volume under the probe [21] was calculated as total haemoglobin, HbT = HHb+HbO. NIRS was sampled at 0.35 Hz.

### Electromyography (EMG)

Surface EMG activity was measured from ECR and trapezius sites as described above. For each site, two self-adhesive, disposable, pre-gelled surface Ag-AgCl electrodes (Ambu neuroline 720 Ag/AgCl, Ambu, Denmark), were attached to the skin in line with the muscle fiber. As stated, the electrodes were placed just under the shield used to house the oxygenation probe. The inter-electrode center distance was ∼20 mm. On the ECR muscle, electrodes were placed perpendicularly under the external rim of the NIRS probe shield and on the trapezius muscle the EMG electrodes were placed perpendicularly to the NIRS probe shield and under the descending lumbar part of the shield. A ground electrode was applied at the level of the C7 vertebrae. Prior to electrode attachment the skin was dry shaved and rubbed with an ether/alcohol mixture.

The EMG signal was amplified and bandpass-filtered (second-order Butterworth 10-1000 Hz) using an 8-channel amplifier (x500) (Octopus AMT-8 – Bortec Electronics). Sampling was done continuously during the experiment using a 16-channel 12-bit A/D converter (CED Mikro 1401, Cambridge Electronics Design, UK) at a rate of 2086 Hz with the Spike 2 software (CED, Cambridge Electronics Design, UK).

### Heart rate variability (HRV)

HRV was measured continuously during all procedures using a bipolar electrocardiogram (EKG). For EKG, two self-adhesive, disposable, pre-gelled surface Ag-AgCl electrodes (Ambu neuroline 720 Ag/AgCl, Ambu, Denmark), were attached at the level of the sixth left rib and the distal end of the sternum. The EKG signal was amplified, filtered, and sampled in the same way as the EMG. Inter-beat intervals (IBIs) from the electrocardiogram were plotted against time for visual inspection, and for semi-automatic editing of abnormal and ectopic beats using linear interpolation.

### Maximal voluntary contractions (MVC)

Three MVCs were performed for 5 s each with a 2 min recovery time between contractions for wrist extension and shoulder elevation, respectively. MVCs were performed using the strain gauge-strap set-up and handle arrangements as described above. Participants were encouraged to perform their best for each MVC. The force signals were amplified with an IPS100C-1 amplifier (BIOPAC, CA, USA) and sampled at 100 Hz by a 12-bit A/D converter, and transferred to the Spike 2 software by the use of a customary script. The maximal value was obtained after smoothing the signal with a moving average window average of 0.1 s for each occasion, and was used to determine the sustained contraction levels for the ECR and TD.

### Sustained isometric contraction

For both the ECR and TD muscles a target level of 15% MVC was determined. Participants performed a sustained isometric extension of the right wrist and elevation of the shoulder at 15% MVC maintained until participants could no longer hold contraction or to an endurance cutting point time set for 12 min. This cut-off time was selected because it was expected to yield physiological responses and induce fatigue, and in a previous study the mean endurance time for shoulder abduction was 10.5 min [22]. Also we did not want to aggravate any of the patients' existing medical conditions.

Due to a small variance in the stop times after the 12 min, we used the time frame of 12 to 13 min. Muscle fatigue was identified if contraction force levels dropped by 20% from the previous set up value of 15% MVC for more than 3 s [23]. Visual feedback of the force was provided to participants throughout contractions by a line representing the target force level on a computer screen in front of them. The fatigue threshold point was not shown at any time. Participants were encouraged to attain target levels as quickly as possible but were not encouraged to maintain target levels throughout the contraction. Participants stopped the contraction when they felt fatigued, force dropped 20% of the set level for more than 3 s or, after 12 min. For those participants that maintained the full duration, at the 12 min cut-off time the investigator casually approached the participant and informed him/her to discontinue. Thus, in this case the endurance time recorded was between 12 and 13 min. The stop point was defined as the endurance time. The participants had no knowledge of the elapsed time during the task. If the investigator stopped the task the participants were not informed about the elapsed time or the reason for stopping.

### Venous occlusion (VO)

Performed for the ECR only, participants were tilted back into a hospital gurney with the arm supported such that the site of the NIRS probe over the ECR was at heart level. For this procedure, after participant positioning and a rest period of 5 min, a pneumatic cuff was placed around the upper arm and rapidly inflated to a pressure 50 mmHg to attain VO and maintained for 1 min. The time to inflation was 3–5 s. VO was used to attain an estimation of blood flow into the tissue by looking at the increase in HbT. At 50 mmHg venous outflow is blocked, while arterial inflow is not affected. Applying VO with NIRS for these purposes has been validated against standard methodologies in previous studies [24]–[26].

### Test contractions (TC)

For both the ECR and TD muscles, test contractions (TC) were performed on four different occasions. For the ECR the TC consisted of an extension of the wrist joint with a strap placed on the dorsal part of the hand with a 1-kg weight on its end. For the TD the TC consisted of a flexion of the glenohumeral joint with the arm extended. For both muscles TC were performed once before the sustained contraction as a baseline value and three times after the contraction, once immediately after and then fifteen minutes and thirty minutes after the end of the sustained contraction. TCs were used to assess myoelectric activity during the recovery period after the isometric sustained contraction.

### Subjective fatigue rate (SFR)

Participants rated muscle fatigue perception with the help of a visual analog scale adapted form of the Borg CR-10 scale [27]. Fatigue perception was rated specifically for the muscle being tested at that time. Participants rated their fatigue on four different occasions before each TC (i.e. before the sustained contraction, immediately after the sustained contraction, 15 min after and 30 min after the sustained contraction). For each rating, participants were asked to mark an “X”, along a line that extended from 0 =  ‘no fatigue at all’ to 10 =  ‘extremely fatigued’.

### Data processing

A mean value of force was calculated for the whole sustained contraction. One-sec averages of the force signal were computed during the whole contraction for each participant, and their standard deviation (Sdf) was calculated. The 1-s averages were also used for the coefficient of variation, which was computed as 100 * Sdf/mean force. Resting StO_2_% was obtained as a mean of the three min rest period prior to the MVCs and expressed the resting values of each subject. NIRS and EMG variables were, due to the large variation in endurance time for sustained contractions, were normalized in time and the total time was expressed as 100% of time. NIRS variable data were interpolated using a linear interpolation function fit in MatLab R2012a (MathWorks, Natick, MA, USA). In this way, additional data points were computed for StO2% and HbT and data were divided into 2 percent time windows for each contraction yielding a total of 50 data points for each variable. For EMG, RMS and MPF were computed as the average of every 2 percent time window for each contraction yielding 50 data points for each variable for each participant. In addition, RMS was normalized as a percentage of its start value. For TCs MPF was computed as one value for the 10 sec contraction times. For the ECR VOs (i.e. including resting blood flow), the slopes of increase in HbT were calculated during the first 45 s of each VO. The rate of increase in HbT (i.e. HbTslope) represented blood flow [24]–[26].

Mean IBIs were calculated from 5 percent of time windows, as resulting in 20 windows irrespective of the recording time. For time domain heart rate variability, the standard deviation of IBIs (SDNN), the square root of the mean squared differences between successive IBIs (RMSSD), and the proportion of differences between adjacent pairs of IBIs>50 ms (pNN50) were calculated [28]. In order to assess the change in RMSSD and pNN50 over time, an overlapping 60-s moving window was used, with each IBI resulting in a new data point. The HRV indices were then averaged for each 5 percent of time segment.

### Statistical analyses

Statistical analyses were performed in SPSS 20.0 (IBM Inc., Chicago, IL, USA). Because there are morphological differences between forearm and shoulder muscles, and that our experimental design did not control for synergistic involvement our purpose was to look at each muscle separately. To analyze differences in mean force, resting StO_2_% and resting ECR blood flow, *t* tests were applied. To analyze differences in endurance time Mann-Whitney tests were used.

For NIRS, EMG and EKG variables during the sustained contraction full factorial repeated measures analyses of variance (ANOVA) were used to assess differences between patients and controls over time. The dependent variables StO2%, HbT, RMS, MPF, IBI, pNN50, RMSSD and SDNN were analyzed in the ANOVA model with time as the categorical independent variable and group (patients and controls) as the between-subjects factor. Group effects were only reported when significance occurred.

For VO, TC and fatigue ratings full factorial repeated measures ANOVAs were used to assess differences between patients and controls over measuring occasions with group (patients and controls) as the between-subjects factor. If the assumption of sphericity was not met, a Greenhouse-Geisser correction was applied. In all tests, *P*<0.05 was considered significant and *P*<0.10 was considered a limit for tendencies. Data are presented in the tables as mean ±SD, and in the figures for the sake of clarity only mean values are shown. For endurance time median times and interquartile ranges (IQR) are presented.

## Results

Table 1 shows personal data for patients and controls. For both the ECR and TD muscles MVC and MTh were not significantly different between patients and controls. ATT was significantly higher for the TD of patients but not for the ECR. For the TD controls presented a higher level of StO_2_% at rest. There were no differences in resting blood flow between patients and controls. Although no significant differences were seen in blood pressure between patients and controls, a tendency for difference was found in diastolic pressure.

### Endurance time

**Figure 2:**
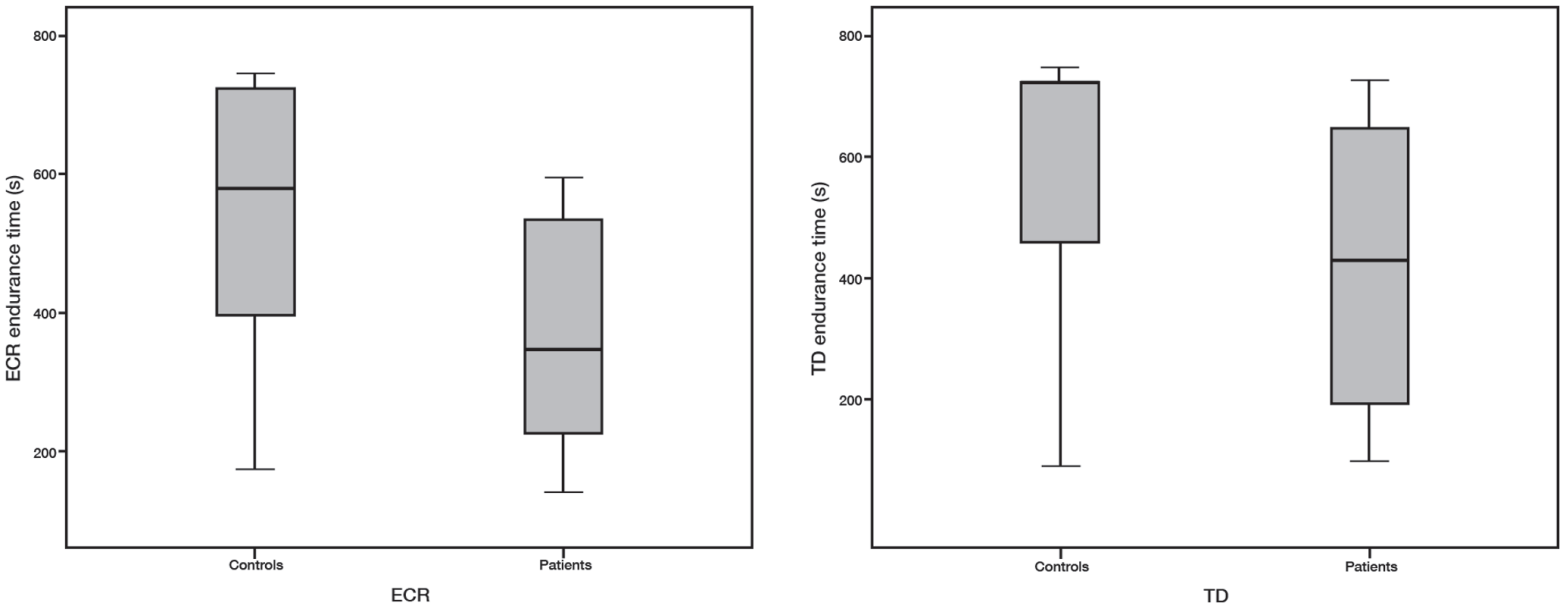
Endurance times. Median endurance times represented by the lines in the boxes for healthy controls and patients with WRMP for the ECR (left) and TD (right). The lower and upper endpoints of the boxes represent the 25^th^ percentiles (lower quartile) and 75^th^ percentiles (upper quartiles), respectively. The distances between these endpoints represent the interquartile ranges. The endpoints of the bars extending out from the boxes represent the minimum and maximum values.

Figure 2 shows the endurance times for patients and controls for the ECR and TD. For endurance time Mann-Whitney tests showed significant differences between patients and controls for both the ECR and TD muscles. For both muscles patients in general reached their endurance times before the cut-off time of 12 min. For the ECR endurance time, the medians were 347 s (IQR = 208.0 – 537.0 s) for patients, and 582 s (IQR = 383–724 s) for controls (*P* = 0.01) with 11 controls (out of 18) reaching the endurance time before 12 min whilst all patients reached the endurance time before 12 min. For the TD endurance times, the medians were 430 s (IQR = 194.0–682. s) for patients, and 723 s (IQR = 437–726 s) for controls (*P* = 0.03) with 7 controls and 12 patients reaching the endurance time before the cut-off time of 12 min.

### Subjective ratings of fatigue

**Figure 3:**
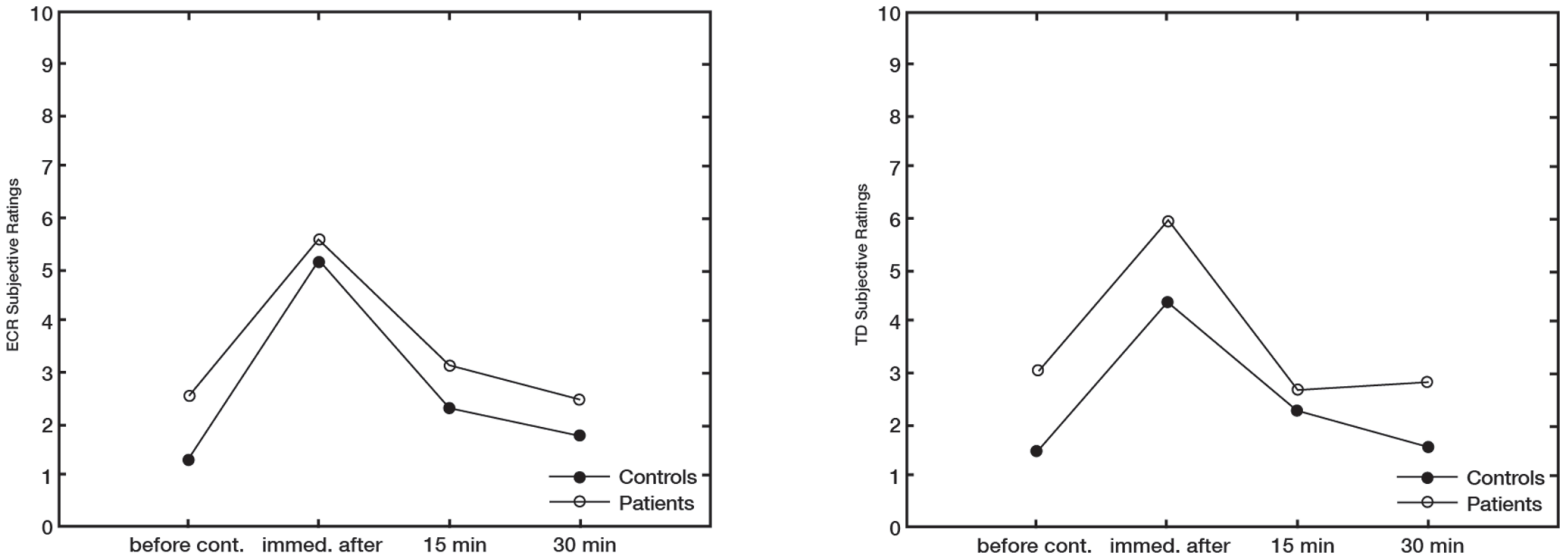
Subjective Fatigue Ratings. Mean changes in subjective fatigue ratings over time for the ECR (left) and TD (right) muscles. Ratings were assessed before the sustained contraction (before cont.), immediately after the sustained contraction (immed. after), and during the recovery period (15 min and 30 min) for patients and controls.

Figure 3 shows the changes for subjective ratings for the ECR and TD over measurements with respect to before the sustained contraction and during the recovery period for patients and controls.

For the ECR subjective ratings there were significant changes over time [F (df 2.07)  = 56.16; *P*<0.001], but these changes were not significantly different between patients and controls [F (df 2.07)  = 1.16; *P* = 0.32]. The general trend, however, was for patients to report higher values than controls.

For the TD subjective ratings there were significant changes over time [F (df 1.84)  = 38.54; *P*<0.001], but these changes were not significantly different between patients and controls [F (df 1.84)  = 1.70; *P* = 0.19]. There was, however, a trend for patients to report higher values than controls.

### Venous Occlusions (VO)

For VO, HbTslopes the ANOVA showed no significant differences over measurements with respect to VO initial and during the recovery period [F (df 2.29)  = 0.24; *P* = 0.81], and no significant differences between patients and controls [F (df 2.29)  = 1.62; *P* = 0.20].

### Test contractions (TC)

For the ECR TCs there were significant differences in the EMG MPF between measurements with respect to TC initial and during the recovery period [F (df 1.96)  = 3.32; *P* = 0.04]. These changes, however, were not significantly different between patients and controls [F (df 1.96)  = 1.97; *P* = 0.15].

For the TD TCs there were no significant differences between measurements with respect to TC initial and during the recovery period [F (df 2.39)  = 0.66; *P* = 0.55], and no significant differences between patients and controls [F (df 2.39)  = 0.84; *P* = 0.45].

### ECR during the sustained contraction

For the ECR force CV, the repeated measures ANOVA showed a significant change over time [F (df 7.56)  = 6.36; *P*<0.001], however, this change was not significantly different between patients and controls [F (df 7.56)  = 1.32; *P* = 0.24].

**Figure 4:**
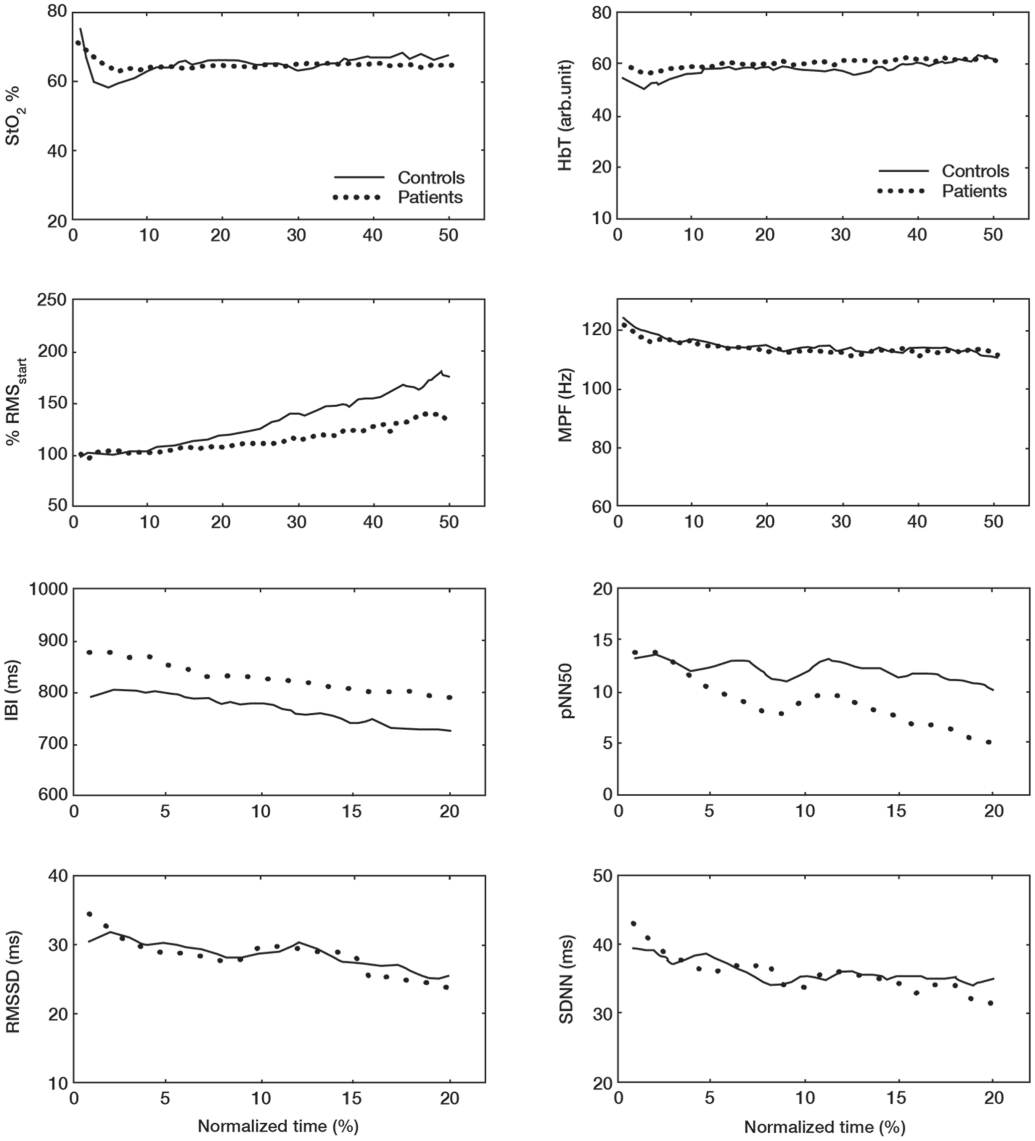
NIRS, EMG and EKG responses for the ECR muscle contraction. Mean changes in StO_2_%, HbT, RMS, MPF, IBI, pNN50, RMSSD and SDNN for the ECR during the sustained contraction.

Figure 4 shows the mean changes in StO_2_%, HbT, RMS, MPF, IBI, pNN50, RMSSD and SDNN for the ECR during the sustained contractions.

For StO_2_% there were significant changes over time [F (df 2.68)  = 4.02; *P* = 0.01], however, these changes were not significantly different between patients and controls [F (df 2.68)  = 1.92; *P* = 0.14]. The general trend of the changes was an initial decrease for both groups with controls showing a larger drop. After this patients remained unchanged throughout the contraction while controls showed an increasing pattern. Also, HbT changed significantly over time [F (df 5.51)  = 7.30; *P*<0.001], but the changes were not significantly different between patients and controls [F (df 5.51)  = 1.32; *P* = 0.26]. As with StO_2_% the general trend was an initial decrease with gradual increase throughout the contraction and a similar pattern as of StO2% for both groups.

RMS increased significantly over time [F (df 1.60)  = 20.19; *P*<0.001], but the responses were not significantly different between patients and controls. There was, however, a tendency for difference between with controls showing a larger increase over time than patients [F (df 1.60)  = 2.72; *P* = 0.09]. MPF decreased significantly over time [F (df 5.85)  = 9.95; *P*<0.001] but there was no significant difference between patients and controls [F (df 5.85)  = 0.55; *P* = 0.76].

For the ECR IBI there were significant changes over time [F (df 4.11)  = 28.30; *P*<0.001] however, these changes were not significantly different between patients and controls [F (df 4.11)  = 0.85; *P* = 0.50]. For pNN50 there were significant changes over time [F (df 2.80)  = 3.53; *P* = 0.02] but changes did not differ significantly between patients and controls [F (df 2.80)  = 1.13; *P* = 0.34]. For RMSSD there were significant changes over time [F (df 3.28)  = 4.87; *P* = 0.002] but these changes were not significantly different between patients and controls [F (df 3.28)  = 0.43; *P* = 0.75]. For SDNN there were significant changes over time [F (df 4.37)  = 3.05; *P* = 0.02] but they were not significant between patients and controls [F (df 4.37)  = 0.51; *P* = 0.75].

### TD during the sustained contraction

For force TD force CV, the repeated measures ANOVA showed a significant change over time [F (df 3.80)  = 2.95; *P* = 0.03], however, these changes were not significantly different between patients and controls [F (df 3.80)  = 1.21; *P* = 0.31].

**Figure 5:**
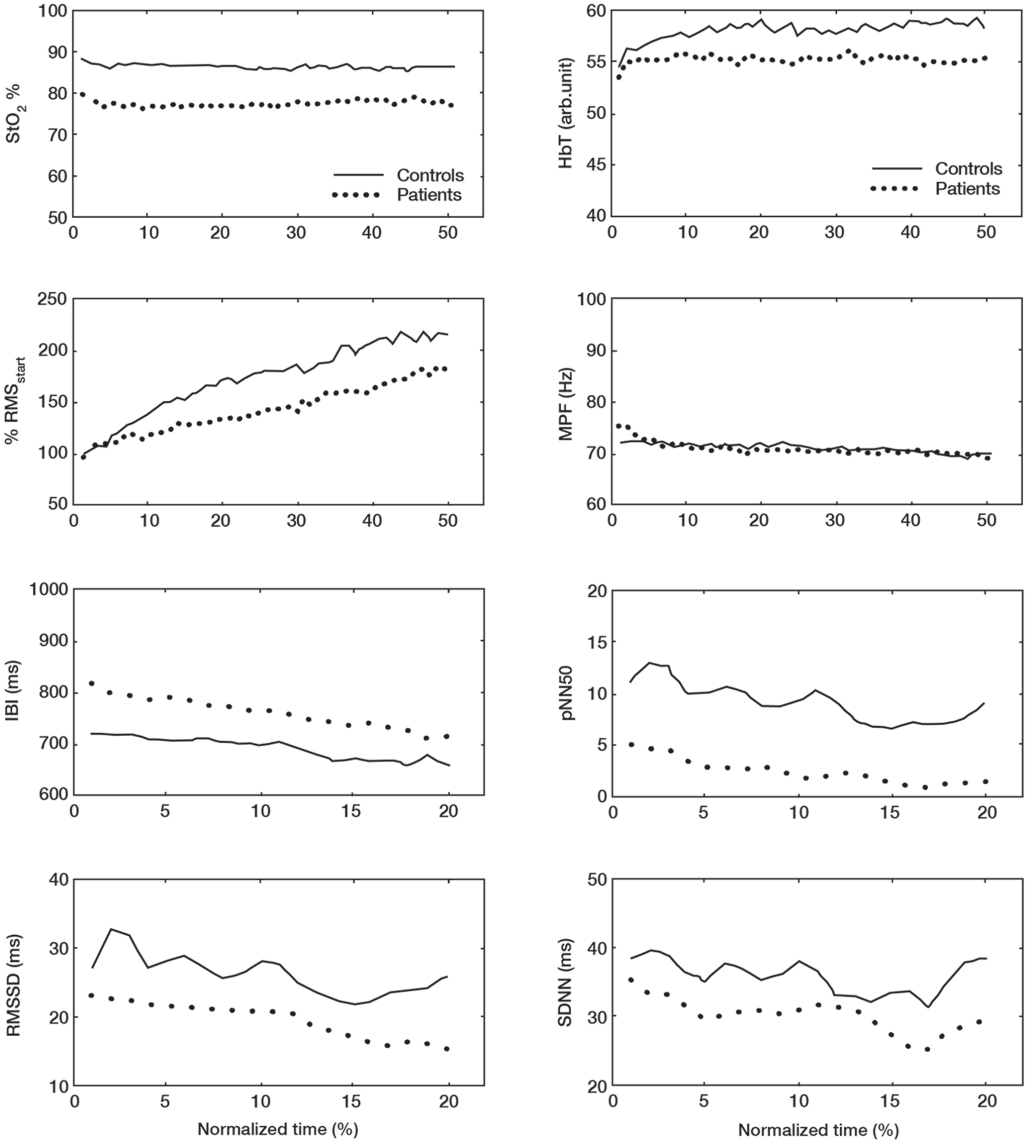
NIRS, EMG and EKG responses for the TD muscle contraction. Mean changes in StO_2_%, HbT, RMS, MPF, IBI, pNN50, RMSSD and SDNN for the TD during the sustained contraction.

Figure 5 shows the mean changes in StO_2_%, HbT, RMS, MPF, IBI, pNN50, RMSSD and SDNN for the TD during the sustained contractions.

StO_2_% did not change significantly over time [F (df 3.43)  = 1.01; *P* = 0.40] and there was no difference between patients and controls [F (df 3.43)  = 1.10; *P* = 0.37]. There was, however, a significant group effect with patients showing a lower StO_2_% value at the start and throughout the contraction [F (df 1)  = 6.49; *P* = 0.02]. HbT did not change significantly over time [F (df 2.36)  = 1.71; *P* = 0.18], and there were no significant differences between patients and controls [F (df 2.36)  = 1.01; *P* = 0.38]. RMS increased significantly over time [F (df 1.50)  = 13.60; *P*<0.001], but there was no significant difference between patients and controls [F (df 1.50)  = 0.78; *P* = 0.46]. MPF decreased significantly over time [F (df 1.83)  = 3.42; *P* = 0.04], but there was no significant difference between patients and controls [F (df 1.83)  = 0.81; *P* = 0.44].

For the TD IBI there were significant changes over time [F (df 3.24)  = 19.11; *P*<0.001] however, these changes were not significantly different between patients and controls [F (df 3.24)  = 1.21; *P* = 0.31]. For pNN50 there were significant changes over time [F (df 3.32)  = 4.67; *P* = 0.003] but changes did not differ significantly between patients and controls [F (df 3.32)  = 0.58; *P* = 0.64], although there was a significant group effect between patients and controls for pNN50 [F (df 1)  = 4.42; *P* = 0.04] with controls showing higher values. For RMSSD there were significant changes over time [F (df 4.31)  = 6.33; *P*<0.001] but these changes were not significantly different between patients and controls [F (df 4.31)  = 0.75; *P* = 0.57]. For SDNN there were significant changes over time [F (df 4.91)  = 3.81; *P* = 0.62] but they were not significant between patients and controls [F (df 4.91)  = 0.71; *P* = 0.75].

## Discussion

There were a greater number of patients that were unable to maintain the 15% MVC for the 12 min allotted time compared to controls. Consequently, the endurance times were significantly shorter for the patients for both muscles. This complies with other studies that have shown earlier fatigue for WRMP subjects than controls during exercise [3], [5], [6], [29]. The magnitude and duration of the sustained contraction were used to allow time for good physiological responses, but not too long such that the physiological responses would be more influenced by time than by group condition. The primary aim of the study was to see if the early fatigue for WRMP patients was associated with hemodynamics and oxygenation. Since we found no significant differences between groups in baseline oxygen saturation (except for the TD) and blood flow, as well as no difference in StO_2_% responses during the contraction (i.e. consumption), we deduce that hemodynamics and/or oxygenation did not underlie early fatigue for our WRMP patients. The significantly lower StO_2_% value for the TD of patients could suggest a reduction in the microcirculation compared to controls. However, the magnitude for the patients was still quite high at 76% (see table 1), and therefore, this difference is unlikely to play a substantial role in the shorter endurance time. Also, our other physiological fatigue indicators – force CV, RMS and MPF, were not different between groups for either muscle. It could be speculated that the fatigue was secondary to sensations of pain experienced by the patients during the contractions. We cannot ascertain this because, unfortunately, we did not inquire about pain either before, during or after the sustained contraction. Taken together our findings could lean toward a central as opposed to peripheral mechanism governing the fatigue.

In addition, while there were overall increases in subjective fatigue when relating before to after the sustained contraction (as expected), there was no significant differences between groups for either muscle. This latter finding is somewhat surprising because one could expect that since patients on average had lower endurance times than controls, they would also have rated significantly higher regarding perceived fatigue, especially immediately post contraction. A significant inverse relation between subjective fatigue ratings and endurance time was reported previously [30], but this was not reflected in our data. Our participants were asked to rate the fatigue specifically directed to the muscle being tested, so the uncoupling between the perceived rating and the sensation of endurance may further strengthen the presumption of a central fatigue mechanism for our data.

An interesting note is that MVCs were not significantly different between groups for either muscle. This is in contrast to several previous studies that reported that patients had a lower MVC than controls [14], [15], [31], [32]. This means that the absolute force level during the endurance test (calculated as a percentage for each individual) was also similar between patients and controls. This brings into the discussion the severity (or lack thereof) for our patient group in comparison to other studies. Our patients were currently working and participated in some type of physical activity at a training facility. Therefore, applying our experimental approach to a more severe patient group may yield different results.

### Group comparisons for NIRS, EMG and EKG

A secondary aim of the study was to compare oxygenation responses for the two groups during the sustained contraction, and to support the discussion with responses in EMG and EKG data. NIRS measurements represent the dynamic balance between oxygen delivery and oxygen consumption (c.f. Ferrari *et al*. [33]) such that a drop in StO_2_% during activity means that more oxygen is being consumed relative to what is being delivered. For studies involving low-level repetitive activity, i.e. dynamic activity, a consistent pattern in StO_2_% has been observed for both healthy subjects and WRMP patients that entail a drop early in the activity that either plateaus or trends upward back toward baseline. For our sustained isometric contraction, StO_2_% data for the ECR displayed this typical pattern with an abrupt drop then a trending increase for healthy subjects, whereas for patients there was an immediate drop that plateaued for the duration of the contraction. This trend agrees with that described by Sjogaard *et al*. [15] for measuring NIRS on the shoulder for patients and healthy subjects performing pegboard work. For TD, however, we found no change in StO_2_% throughout the contraction. Of particular relevance to the study was that the ANOVA revealed that responses in StO_2_% as well as HbT (blood volume) were not significantly different between groups (i.e. no interaction) for either muscle. This agrees with some previous data [16], but is in contrast to others [14]. In the latter study, the authors report differences in oxygen consumption and not StO_2_% changes, and the differences are measured after, not during, the contraction. We reiterate that our data suggested that muscle oxygenation did not contribute to the differences we found for endurance times between patients and controls.

We found no difference between healthy subjects and WRMP patients in forearm blood flow before the sustained contraction or during the recovery period (i.e. no significant group differences in ECR HbTslope). This finding is in contrast to Oskarsson *et al*. [34] using Laser Doppler flowmetry, and Brunnekreef *et al*. [35] using a flow mediated dilation technique, but in somewhat agreement with Brunnekreef *et al*. [14] using NIRS. Regarding the former studies, it may be easy to account for discrepancies between those results and ours based on different techniques. This could open discussion about the sensitivity of different techniques in detecting forearm blood flow. Of course, the patient groups were also different between studies. In the previous NIRS study by Brunnekreef *et al*. [14], the authors found no group difference in baseline blood flow, as did we, but reported significantly higher values for controls following intermittent exercise consisting of varying loads (10%–40% MVC). It is difficult to equate their exercise protocol to our sustained 15% MVC contraction, which may partly explain differences between studies. It is worthy to comment on the fact that, regardless of group, there was no change in HbTslope following the sustained contraction. This was surprising since it is a normal physiological response that blood flow would increase following a contraction. A previous NIRS study using a similar protocol (15-min at 10% MVC) showed increased HbTslope post contraction [10] for the flexor digitorum superficialis muscle. A discrepancy between studies may be due to the different muscles used.

Our EMG-RMS and MPF data indicate that the activity for both muscles throughout the sustained contraction were as expected physiologically. It is surprising then that there was no change in StO_2_% for TD considering that the muscle was active throughout, which likely required metabolic activity. The increase in RMS and decrease in MPF are typical responses of fatigue development [9], [36]. While there were no significant differences in either RMS or MPF responses between patients and controls, there was a tendency for differences for the ECR with patients showing a lower RMS than controls. We had initially presumed that this tendency could be due to the different endurance times since more control subjects than patients maintained the contraction for the full duration. However, when we tested this statistically we found that this was not the case. It is important to point out that Schulte *et al*. [32] found a significant lower EMG RMS for patients compared to controls during a 6 min sustained contraction.

Test contractions eliciting MPF responses were intended to monitor whether fatigue was present during the recovery period. A significant effect (recovery vs. initial) was noted for the ECR, but not for the TD; however, there was no difference between patient and controls for either muscle. The use of EMG to assess muscle fatigue in the context of our experimental design has been questioned, thereby giving preference to more sensitive measurements such as mechanomyography and/or electrical stimulation. Both of these methods have shown long-lasting fatigue following low-level sustained contractions [9], [37]. This brings into question whether our data, in comparing groups, would have been different if we had used mechanomyography or electrical stimulation.

A restriction in blood flow could critically influence the development of muscle fatigue even at low contraction force levels [38]. It has also been suggested that restrictions in the circulation may disturb muscle homeostasis and thus contribute to the development of WRMP [39]. Moreover, it has been also suggested that the autonomic nervous system could further contribute to the restriction in blood flow in WRMP [40]. In the present study, the patient group had a marginally increased blood pressure at rest, and reduced pNN50 during the sustained contraction for the TD muscle compared to controls. We could, therefore, argue that a mild deviation in basal autonomic activity may exist. However, in contrast to previous studies [41], [42], we did not find evidence for differences in autonomic reactivity between patients with WRMP and controls.

In all NIRS studies a factor to consider is the influence of ATT, which is known to influence the NIRS signal during exercise [43], [44]. For the ECR there were no significant differences in ATT between patients and controls, and for the TD there was a significant difference between patients and controls with patients showing a higher ATT value. The mean value for the ATT in the TD for patients is 5.97 mm vs. 4.98 mm for controls and the NIRS light penetration depth is considered to be 12.5 mm (half the size of the probe) [45]. We believe thus that enough depth of muscle tissue was measured and our results are not influenced by differences in ATT. A second concern may be the dilution of data caused by our analysis since NIRS data points were interpolated. Data, however, were statistically checked without interpolation and the initial drop in StO_2_% was not different between controls and patients.

In summary, patients with WRMP fatigued earlier than healthy controls during low-level sustained contractions for the ECR and TD muscles. However, we found no differences between groups in baseline oxygen saturation (except for the TD) and blood flow, as well as no difference in StO_2_% responses (i.e. oxygen consumption) during the contraction, therefore, our results indicate that hemodynamics and/or oxygenation do not underlie early fatigue for WRMP patients. These data taken together with our other physiological data, i.e. EMG and EKG, suggest that early fatigue observed for WRMP patients is governed by a central as opposed to peripheral mechanism.
